# Factors Affecting Balance Performance in Adolescents

**DOI:** 10.3390/children11040436

**Published:** 2024-04-05

**Authors:** Milena Kovačević, Rastislava Krasnik, Aleksandra Mikov, Darko Mikić, Jelena Zvekić-Svorcan, Dragana Vukliš, Dajana Dedić Novaković, Marina Đelić

**Affiliations:** 1 Faculty of Medicine, University of Novi Sad, 21000 Novi Sad, Serbia; rastislava.krasnik@mf.uns.ac.rs (R.K.); aleksandra.mikov@mf.uns.ac.rs (A.M.); jelena.zvekic-svorcan@mf.uns.ac.rs (J.Z.-S.); dragana.vuklis@mf.uns.ac.rs (D.V.); 1414d20@mf.uns.ac.rs (D.D.N.); 2 Faculty of Medicine, University of Belgrade, 11000 Belgrade, Serbia; 3 Institute for Child and Youth Health Care of Vojvodina, 21000 Novi Sad, Serbia; 4 Pathology and Forensic Medicine Institute, Medical Faculty of the Military Medical Academy, University of Defence, 11000 Belgrade, Serbia; darenije@gmail.com; 5 Special Hospital for Rheumatic Diseases, 11000 Novi Sad, Serbia; 6 Oncology Institute of Vojvodina, 21208 Sremska Kamenica, Serbia; 7 Institute of Medical Physiology “Richard Burian”, Faculty of Medicine, University of Belgrade, 11000 Belgrade, Serbia; marina.djelic@med.bg.ac.rs

**Keywords:** balance, adolescence, anthopometry, physical activity

## Abstract

(1) Background: The influence of different factors on balance in adolescence is assessed by conducting functional balance tests that examine its different components. (2) Materials and methods: The study sample comprised 110 healthy adolescents of both sexes, aged 12–18 years. Single Leg Stance with Eyes Open (SLS-EO) and Eyes Closed (SLS-EC) tests were conducted to evaluate static balance, whereas the Functional Reach Test (FRT) and Lateral Reach Test (LRT) were performed to establish functional stability limits. The influence of sex, age, demographic factors, anthropometric characteristics, participation in sports activities, and trunk extensor muscle endurance (Biering–Sorensen test) on balance performance was determined through correlational and univariate linear regression analyses. (3) Results: Older age (Beta [β] = 0.247; 95% CI [0.75, 5.20]; *p* < 0.01) and better trunk extensor muscle endurance (β = 0.224; 95% CI [0.015, 0.13]; *p* < 0.05) were significant predictors of the SLS-EO results, while younger age (β = −0.219; 95% CI [−1.32, −0.11]; *p* < 0.05) and higher muscle percentage (β = 0.237; 95% CI [0.06, 0.48]; *p* < 0.05) emerged as significant predictors of LRT performance, and greater bone mass was a significant predictor of FRT results (β = 0.444; 95% CI [3.62, 8.17]; *p* < 0.01). However, none of the independent variables was a statistically significant predictor of the SLS-EC results. (4) Conclusions: The current study found that age, trunk extensor muscle endurance, muscle percentage, and bone mass are significant predictors of different balance components, suggesting that balance is task-specific.

## 1. Introduction

Balance, which is defined as the ability to maintain the body’s line of gravity above the support surface, is the basis for all voluntary motor skills [1,2]. The ability to maintain balance is crucial for optimal motor control, as well as for performing activities of daily life, and is compromised by the sedentary lifestyle that prevails in modern society [3]. If balance is impaired at an early age, it can adversely affect the child’s ability to acquire complex motor skills, and therefore to engage in sports activities safely [4,5]. For these reasons, balance assessment in children is extremely important, and is usually performed by physical therapists and sports coaches to determine a child’s ability to move safely in different environments or to resume sports participation after an injury [6].

Various methods can be used to assess balance, including functional balance tests and balance scales, or more objective technologies such as static and dynamic posturography [7]. As balance comprises multiple components and is the result of interactions among several systems in a constantly changing environment, no single balance test reflects the entire concept of balance [8]. Therefore, it is advisable to perform several balance tests focusing on its different components [2]. In clinical balance tests, static stability, dynamic stability, functional stability limits, and anticipatory postural control are the most frequently evaluated balance components [8]. When interpreting the obtained findings, it is important to consider the factors known to influence balance performance in children, such as age, gender, height, body mass, foot dimensions, dominant leg, level of physical activity, and prior lower limb injuries [9], along with task-related and environmental factors [10].

In the extant literature and clinical practice, it is considered that children develop adult-like balance performance in the age range of 7–10 [11]. However, as a part of their meta-analysis, Schedler et al. compared the balance performance in children (aged 6–12 years) with that of adolescents (aged 13–18 years). These authors concluded that improvements in balance performance continue until late adolescence due to neurological maturation and should thus be differently trained during childhood and adolescence [12]. Adolescence is a period in the development of the human body during which major changes occur in height, body mass, and body composition. A large number of children start to engage in various sports activities in this period [13] and thus acquire better balance performance than their sedentary peers [14,15]. However, in making this correlation, authors of extant studies failed to account for the duration and frequency of sports participation and their impact on balance. Research on the influence of trunk extensor muscle endurance on balance performance is also limited, and in the few studies in which this connection was examined, the sample included not only adolescents but also older individuals, all of whom were male [16,17].

These gaps in the extant literature have motivated the present study, the aim of which was to conduct a battery of functional balance tests to examine the influence of various factors such as sex, age, demographic and anthropometric characteristics, duration of sports participation, weekly frequency of sports sessions, and trunk extensor muscle endurance on the different balance components of healthy adolescents.

## 2. Materials and Methods

This cross-sectional study was conducted on a representative sample of 110 healthy adolescents of both sexes, aged 12–18 years, who were randomly selected from primary and secondary schools within the territory of the city of Novi Sad, Serbia, during May 2023. The sample size was calculated by an online calculator (Raosoft, 2024). For the current study, a minimum number of 106 samples was required considering a 90% confidence level and 8% marginal error. Due to the nature of data collection, additional samples were enrolled to compensate for potential missing or unintended error.

Prior to commencing the study, participating children gave their verbal consent, and their parents/guardians signed an informed consent form. The study was conducted in accordance with the Declaration of Helsinki, and the research protocol was approved by the Ethics Committee of the Faculty of Medicine, University of Belgrade (number 17/V-5).

When assessing the children’s eligibility for participation, the following exclusion criteria were applied: presence of neurological and neuromuscular diseases, >2 cm difference in the lower limb length, previous surgical treatment of spinal deformity, vision and hearing problems and/or middle ear infections in the preceding six months, fractures or other musculoskeletal injuries of the lower limbs in the last six months, insufficient mental development, headaches, tinnitus, nausea, and feeling of weakness 24 h prior to and/or at the time of examination.

At the start of the study, all participants completed the following questionnaires:

1. A sociodemographic questionnaire created specifically for research purposes, probing into their gender, age, place of residence, educational attainment of both parents, academic performance, and dominant hand.
2. Physical activity questionnaire designed by the researcher for the purposes of the study, containing questions related to playing sports, duration of sports participation (in months), number of hours per week dedicated to sports activities, and regular attendance at physical education classes.

Next, all subjects took part in a physical examination as a part of which their body height, body mass, fat percentage, muscle percentage, and bone mass (the mineral component) were measured using an electronic digital scale (Beurer^®^ BF 400, Beurer GmbH, Ulm, Germany) that works on the principle of bioelectrical impedance. Body mass and body height values were subsequently used to determine the body mass index (BMI) [18].

To examine the influence of trunk extensor muscle endurance on balance performance in adolescents the Biering–Sorensen test was conducted. It has been documented as valid, reliable, safe, practical, responsive, easy to administer, and cost-effective [19]. When performing this test, the subject lies in a prone position on the examination bed so that the upper edge of the iliac bone is level with the edge of the bed. The lower body is stabilized to the bed with three straps, fastened around the pelvis, knees, and ankles, respectively. The subject should maintain the upper part of the body in a horizontal position while the arms are crossed on the chest, and the length of time (in seconds) that the subject can maintain the given position is measured using a stopwatch. The test terminates when the subject can no longer maintain the given position or after 240 s [20].

### 2.1. Balance Performance Assessment

Balance performance was measured through functional balance tests, because they are simple and quick to perform, do not require expensive equipment, and can predict the risk of falling [21]. The tests were conducted in a quiet room, with a good light source, and in a safe place to prevent the risk of falls or injuries during testing. Subjects were instructed to remove their footwear and were allowed to make a trial attempt to gain familiarity with the procedure, after which another attempt was made for the purposes of data collection.

Single Leg Stance Test with Eyes Open (SLS-EO) and Single Leg Stance Test with Eyes Closed (SLS-EC) were used to evaluate static balance. Single Leg Stance Test is a valid tool for examining static balance in children [22], with high intraobserver and interobserver reliability (r = 0.99) and test–retest reliability (r = 0.77) [23]. When performing these tests, the subject stands on one leg, while the other leg is raised and does not touch the standing leg. The arms are crossed on the chest. The researcher uses a stopwatch to measure (in seconds) how long the subject can stand on one leg. The test terminates when the subject moves the arms from the starting position, moves the raised leg, moves the standing leg to maintain balance, opens the eyes in the case of SLS-EC [24], or when the limit set at 60 s is reached [23]. Both tests were performed on both legs, and the better result was used in the subsequent analyses.

The Functional Reach Test (FRT) and Lateral Reach Test (LRT) were conducted to examine functional stability limits [25,26]. FRT is a valid test as it correlates with center of pressure excursions (r = 0.71) and has excellent reliability (ICC = 0.92), as demonstrated by Duncan et al. [26]. LRT is a valid clinical measurement for medio-lateral stability as it significantly correlates with both laboratory measurements of reach (r = 0.650) and center of pressure stability limits (r = 0.331), with high test–retest repeatability (ICC = 0.999) [27].

In the FRT, the subject stands parallel to the wall, with the arm next to the wall in a 90° shoulder flexion position, with the elbow in full extension, the forearm in pronation, the wrist in a neutral position, and the fingers extended. The opposite arm was maintained in a neutral position, relaxed by the side of the body. The measuring tape is placed at the acromion level and the value reached by the tip of the middle finger of the outstretched hand is recorded. The subject is then instructed to reach forward with an outstretched hand as far as possible, without moving their feet, making a lunge, or holding on to the wall or the examiner, aiming to hold this position for 3 s. The value reached by the tip of the middle finger in this position is recorded, and the difference between this and the initial value is taken as the test result.

When performing the LRT, the subject stands parallel to the wall with their back to it, with one arm in a 90° shoulder abduction position, elbow in full extension, forearm in pronation, wrist in neutral position, and fingers extended, while the other arm is next to the body. The measuring tape is placed at the acromion level and the value reached by the tip of the middle finger of the outstretched hand is recorded. Next, the subject is instructed to reach with the outstretched hand to the side as far as feasible, while keeping their feet in the same position, not making a lunge, not clinging to the wall or the examiner, aiming to hold this position for 3 s. The examiner records the distance reached by the tip of the middle finger in this position, and the difference between this value and the initial measurement represents the test result.

Both FRT and LRT were performed with both hands, and the greater value was used in the analyses.

### 2.2. Statistical Analysis

The normality of the distribution was examined using the Shapiro-Wilk test and Kolmogorov-Smirnov test and they showed that the distribution of numerical variables deviated statistically significantly from normal distribution. Skewness and Kurtosis values also showed deviation from normal distribution. Because of that, the median (Mdn) and interquartile range (IQR) were calculated and reported. As the distribution of numerical variables deviated statistically significantly from normal distribution, the non-parametrical Mann-Whitney U test and the Kruskal Wallis test were conducted to test differences in the variables of interest and the relationships were assessed via the Spearman’s correlation coefficient (rho). Univariate linear regression analysis was performed to evaluate the predictive properties of independent variables. A probability level of *p* ≤ 0.05 was considered statistically significant. All statistical analyses were carried out using the SPSS for Windows, ver. 24.0 (IBM Corp., Armonk, NY, USA).

## 3. Results

The study involved 110 adolescents of both sexes aged 12–18 years (Mdn = 15.6, IQR = 2.7), 42.7% of whom were male. There was a statistically significant positive correlation between the subjects’ age and their SLS-EO performance (rho = 0.231; *p* < 0.05), while the correlation between age and LRT performance was statistically significant and negative (rho = −0.221, *p* < 0.05). Other sociodemographic data are presented in Table 1.

When responding to the physical activity questionnaire, 76.4% of the participants indicated that they play sports, and 97.3% stated that they regularly attend physical education classes. Adolescents who play sports exhibited superior SLS-EO test performance compared to those who do not (Mdn = 60.0, IQR = 22.0 vs. Mdn = 44.0, IQR = 42.0; *p* = 0.006). A statistically significant difference in the SLS-EC test results was also noted between adolescents who play sports and those who do not, with a higher value recorded for the former group (Mdn = 11.0, IQR = 12.0 vs. Mdn = 5.0, IQR = 9.7; *p* = 0.038). The duration of sports participation and the weekly frequency of sports sessions were not statistically significantly correlated with balance performance (Table 1).

Anthropometric characteristics of the sample are presented in Table 1. Body height was found to be in a statistically significant positive correlation with the FRT (rho = 0.438; *p* < 0.01) and LRT (rho = 0.288; *p* < 0.01) results, while such a correlation was noted only for body mass and FRT performance (rho = 0.364; *p* < 0.01). The BMI was not statistically significantly correlated with balance performance. The muscle percentage was in a statistically significant negative correlation with the SLS-EC test results (rho = −0.198, *p* < 0.05), while being positively correlated with the LRT performance (rho = 0.228, *p* < 0.05). Finally, bone mass was statistically significantly and positively correlated with the results achieved on both FRT (rho = 0.427, *p* < 0.01) and LRT (rho = 0.211, *p* < 0.05).

The average performance on balance tests in the entire sample and in relation to the age group are presented in Table 2. A statistically significant difference was noted in the SLS-EO test results between subjects aged 12–15 years (Mdn = 44.0 s, IQR = 40.5) and those aged 15–18 (Mdn = 60.0 s, IQR = 15.5), *p* = 0.002. Younger adolescents (Mdn = 28.0 cm, IQR = 5.5) outperformed their older peers (Mdn = 25.0 cm, IQR = 7.0), *p* = 0.007, on the LRT.

Univariate linear regression analysis was conducted to determine whether sex, age, sports participation duration, weekly sports session frequency, BMI, fat percentage, muscle percentage, bone mass, and trunk extensor muscle endurance are statistically significant predictors of balance performance. Both age (Beta [β] = 0.247; 95% CI [0.75, 5.20]; *p* < 0.01) and trunk extensor muscle endurance assessed by the Biering–Sorensen test (β = 0.224; 95% CI [0.015, 0.13]; *p* < 0.05) were shown to be statistically significant predictors of the SLS-EO test performance. These results indicate that, in adolescence, older age and greater extensor muscle endurance contribute to greater balance, as measured by the SLS-EO test. Bone mass emerged as the only statistically significant predictor of FRT results (β = 0.444; 95% CI [3.62, 8.17]; *p* < 0.01), whereas greater muscle percentage (β = 0.237; 95% CI [0.06, 0.48]; *p* < 0.05) as well as younger age (β = −0.219; 95% CI [−1.32, −0.11]; *p* < 0.05) were associated with better performance on the LRT test. No independent variable was identified as a statistically significant predictor of SLS-EC performance (Table 3).

## 4. Discussion

As a part of this study, a battery of functional balance tests focusing on different static balance components and functional stability limits were conducted to evaluate the balance in healthy adolescents aged 12–18 years. This multifaceted approach was adopted, given that balance is task-specific, and performance on a certain test is not predictive for other types of balance [28]. Therefore, a range of tests is needed to assess different types of balance. Sociodemographic and physical activity-related factors influencing performance on these tests in adolescents were also examined.

The average performance of all study participants on the SLS-EO test was Mdn = 60.0 s (IQR = 28.0), while they achieved Mdn = 10.0 s (IQR = 12.2) on the SLS-EC test. As different cut-off limits were used in other studies based on these static balance tests [6,9,23], a comparison of the findings obtained in the present study with those reported in the literature is difficult. According to the published data, children over 10 years of age can balance on one leg for 53–103 s [6,23,29]. Guided by this range, as well as other studies in which the same cut-off time was adopted for the SLS-EO and SLS-EC tests [10,23], we adopted 60 s for both static balance tests in our study. On the other hand, in their research, Condon and Cremin used a cut-off time of 120 s, and demonstrated that children older than 12 years achieved a median of 120 s on these tests [6]. In their study involving adolescents aged 14–19 years, Emery et al. used an even longer cut-off time of 180 s, and reported a median of 26.43 s for the SLS-EC test, which is slightly higher than the comparable result in our study [9]. The average FRT score in our study was Mdn = 39.0 cm (IQR = 7.0), while Mdn = 26.0 cm (IQR = 6.2) was calculated for the LRT. Both values are slightly higher than those related to adolescents in India, Saudi Arabia, and Turkey, but this difference is expected considering that, in these studies, the tested children were aged below 12 years [25,30,31].

Our analyses further revealed that the age of the examinee is in a statistically significant positive correlation with the SLS-EO test result, and in a negative correlation with the performance on the LRT, while no significant correlation was found between age and the achievements on other tests conducted as a part of this research. In addition, older adolescents (aged 15–18 years) achieved statistically significantly higher performance on the SLS-EO test compared to their younger peers (aged 12–15 years). On the other hand, the LRT results were statistically significantly higher among younger participants compared to those older than 15 years. The age groups were determined based on whether children attend primary or secondary school because this is recognized in the literature as a transitional phase that poses a risk for the development of poor lifestyle habits (sedentary behavior, unhealthy diet, increased screen time) [32]. Age proved to be a statistically significant predictor of both SLS-EO and LRT test performance in our cohort, concurring with the assertion made in several studies that balance performance in youth improves with age [6,12,33]. Following their meta-analysis of 21 studies, Schedler et al. concluded that 13- to 18-year-olds have better balance (static balance in particular) compared to 6- to 12-year-olds [12]. Likewise, Condon and Cremin, whose analyses focused on children under the age of 15, found an improvement in static balance in older children [6]. Neurological maturation, but also greater muscle strength and more developed attention skills, contribute to a better ability to maintain balance in older children [12]. Nevertheless, the negative correlation of age with LRT results and better performance on LRT in the younger group of adolescents that took part in our study is noteworthy. As biomechanical factors, such as reach strategies and range of motion in the ankle joint, can also affect the FRT and LRT performance, these are the possible reasons for this finding. As hypermobility is more commonly present in younger compared to older children and decreases with age [34], this could have influenced better lateral reaching in the younger group. During LRT, visual feedback is limited because the subject must not rotate the head and needs to maintain a fixed forward gaze when reaching to the side [25,35]. As younger children rely more on vision when maintaining balance than older children [33], they might have used additional reaching strategies to achieve a better result, which would have contributed to their superior performance relative to their older peers. Accordingly, the strategies that the examinees use during FRT and LRT should also be analyzed as they may influence the results.

Most of the participants in our research were involved in sports, and almost all regularly attended physical education classes. On the SLS-EO, as well as on the SLS-EC test, respondents who play sports had a higher score than those who do not. Better balance abilities of children who are physically active compared to those who are not have been reported by other authors [14,15,36]. Based on their results, García-Soidán et al. concluded that engaging in physical activity can influence more effective balance control, but cannot reverse the impact of neurophysiological factors that are determined by gender [36]. Regarding the duration of sports participation and the weekly frequency of sports sessions, our analyses did not establish significant correlations between these parameters and balance performance, concurring with the results reported by Emery et al. [9]. Likewise, García-Soidán et al. failed to establish a relationship between the number of days per week dedicated to physical activity and balance parameters, and concluded that proper development of postural control does not require a certain minimum dose of physical activity [36]. Additionally, Andreeva et al., in their study, did not establish a correlation between the level of sport performance and postural stability [37]. On the other hand, Hahn et al. reported a positive correlation between the SLS-EO and SLS-EC test results and time spent in basketball training, while this correlation was negative for swimming [38]. Various studies have shown that the type of sport practiced can influence postural stability [37,39]. For example, Trajković et al. found among athletes from seven different sports that dancers demonstrated significantly better stability on one leg compared to others [39].

Postural control is influenced by body dimensions and composition, especially in tests without visual control and, according to Alonso et al., body height is the anthropometric variable that most strongly affects postural sway [40]. The results yielded by our study show that body height has a statistically significant positive correlation with FRT and LRT results, while body mass has a statistically significant positive correlation with FRT only. The findings pertaining to FRT and LRT are supported by those reported by other authors, although their cohorts of interest were younger children (aged 6–12 years) [25,30,31]. Yuksel et al. and Emara et al. also reported results consistent with our findings related to the link between FRT performance and body mass, while Yuksel et al. also established a correlation between body mass and LRT performance [30,31]. On the other hand, Emery et al. found no correlation between the performance on the SLS-EC test and body height or body mass [9], concurring with our findings.

Our analyses further demonstrated that BMI is not statistically significantly correlated with balance performance in our cohort of 12- to 18-year-olds, nor is it a significant predictor of performance on any of the tests used. According to several studies, obese children are less capable of maintaining balance than children of normal weight [41,42]. On the other hand, when Rusek et al. tested the balance of 1137 children aged 7–15 years on the Zebris stabilometric platform, they found that children with a higher BMI had better balance [43]. While this result may seem counterintuitive, the BMI reflects only the relationship between body height and body mass, without accounting for the body composition (in terms of the lean and fat body tissue percentage). Accordingly, we also analyzed the correlation between balance performance and the percentage of fat, the percentage of muscle, and bone mass (the mineral component) in our subjects, as well as assessed their predictive value by conducting univariate regression analysis.

We found that, in our subjects, body fat percentage did not correlate with or predict any of the balance test scores. On the other hand, muscle percentage had a statistically significant negative correlation with the SLS-EC test performance, and a positive correlation with the LRT results, and was a significant predictor of the participants’ achievement on this test. In their study, Rusek et al. found that greater muscle mass significantly correlates with some parameters measured on the Zebris system that are indicative of better balance maintenance [43]. Similarly, Alonso et al. established a positive correlation of lean mass (including muscle mass) with medio-lateral stability [40], providing support for our results regarding the LRT, which is a clinical test for medio-lateral stability. The negative correlation between muscle percentage and the SLS-EC test performance in our cohort could be attributed to the fact that boys have a significantly higher muscle percentage in their body composition compared to girls [43], and in our study, girls slightly outperformed boys on this test. Our analyses further indicate that bone mass has a statistically significant positive correlation with the FRT and LRT results, and was the only statistically significant predictor of FRT performance. Although in their study, Alonso et al. focused on adults, they also found a significant positive correlation between bone mass and postural control, which is in line with our results [40].

According to Helbostad et al., trunk extensor muscle fatigue affects somatosensory processes, leading to poorer balance and coordination [44]. Because of that, we wanted to examine the influence of the results of the Biering–Sorensen test on balance performance. In our research, in addition to age, trunk extensor muscle endurance assessed by the Biering–Sorensen test proved to be a statistically significant predictor of the SLS-EO test performance. These results are supported by those reported by Abaraogu et al., who found a significant positive correlation between static balance and trunk extensor muscle endurance assessed by the same test in male subjects aged 13–25 years [17]. Moreover, Barati et al. showed in their study that the endurance of the extensor muscles is a significant predictor of students’ ability to maintain static balance [16]. Additionally, it has been shown that the strength of the trunk muscles, especially the extensors, affects balance in children with certain types of disabilities [45]. These assertions suggest that people who have problems with balance control could benefit not only from exercises specifically designed for balance enhancement, but also from those aimed at increasing trunk muscle endurance which, according to Barati et al., could lead to 30% improvement in static balance [16].

When interpreting the results reported here, the limitations of our research should be considered. First, the sample size was relatively small in relation to the age range of our cohort. Second, in the FRT and LRT, subjects were instructed to reach forward and sideways as far as possible without losing balance, but no instructions were given regarding the strategy (only the ankle or hip) they could adopt, which could have influenced the results. Third, we did not consider upper limb length when performing FRT and LRT which could also influence the results, as some authors found correlation between them [25,46]. Next, we did not use a validated physical activity questionnaire for the pediatric population, which could have provided us with more detailed data on physical activity. Moreover, considering that the median result on the SLS-EO test was 60 s, a higher percentage of respondents reached the set cut-off limit, suggesting that a longer cut-off time might have been more beneficial, potentially leading to different results. Furthermore, the results of the trunk extensor muscle endurance test did not show a significant correlation with static balance performance. However, the results of the regression analysis indicate that it is a significant predictor of static balance (we have considered the results of the regression analysis to be superior), which calls into question the objectivity of this test. Additionally, although environmental factors are known to influence balance control, these extraneous variables could not be fully controlled in our study. Finally, we only considered the chronological age of the children, but not their biological age and the effect of maturation. Since girls enter puberty earlier than boys, we must not overlook the potential impact of this study limitation on the results.

Nonetheless, our research has many strengths as well, including the adoption of multiple balance tests that examine its different components, allowing us to gain a more comprehensive picture of balance performance in adolescents. Moreover, all conducted tests are inexpensive and easy to use, and can be performed on cohorts of any age. As several factors were assessed for their influence on test performance, this is an additional contribution to the extant literature, but also to clinical practice, as the results reported here can serve as normative data when testing adolescent population.

## 5. Conclusions

Our study shows that the examined factors affect the specific components of balance differently, which indicates that balance is task-specific. In adolescents, older age and greater trunk extensor muscle endurance are significant predictors of the Single Leg Stance Eyes Open Test performance, while younger age and a higher muscle percentage are significant predictors of the Lateral Reach Test results, and greater bone mass is a significant predictor of the achievement on the Functional Reach Test. It is important to assess balance performance in the adolescent population and be aware of factors that may influence it. This can aid in better guiding adolescents towards sports activities aligned with their balance abilities and in designing physical education classes that consider these capabilities. The tests used in this study should be more integrated into everyday practice of healthcare professionals working with adolescents, as well as their sports coaches, to reduce the risks of injuries related to poor balance. Future studies should focus on monitoring balance over time in the adolescent population, considering not only chronological age but also the biological age’s impact on balance performance.

## Figures and Tables

**Table 1:** Sociodemographic characteristics, anthropometric characteristics, and balance performance of the study subjects.

	All Participants (N = 110) (%)	SLS-EO (s)	SLS-EC (s)	FRT (cm)	LRT (cm)
Sex, (*p* Value, Partial Eta^2^)		0.277 ^a^ (0.007)	0.295 ^a^ (0.016)	0.141 ^a^ (0.012)	0.082 ^a^ (0.016)
Female	63 (57.3%)	60.0 (22.7) ^d^	11.0 (11.5) ^d^	38.5 (7.0) ^d^	25.0 (7.2) ^d^
Male	47 (42.7%)	60.0 (35.2) ^d^	9.0 (13.25) ^d^	39.0 (9.0) ^d^	27.0 (7.5) ^d^
Age (years)	15.6 (2.7) ^d^	0.231 (0.06–0.43) *^c^	−0.034 (−0.01–0.15) ^c^	0.023 (−0.46–0.06) ^c^	−0.221 (−0.73–0.19) *^c^
Place of residence, (*p* Value, Partial Eta^2^)		0.295 ^a^ (0.003)	0.278 ^a^ (0.023)	0.262 ^a^ (0.011)	0.102 ^a^ (0.024)
Rural	18 (16.4%)	60.0 (18.2) ^d^	12.0 (19.2) ^d^	37.5 (6.5) ^d^	24.0 (6.5) ^d^
Urban	92 (83.6%)	60.0 (31.0) ^d^	9.5 (12.0) ^d^	39.5 (7.2) ^d^	26.0 (7.2) ^d^
Mother’s educational attainment, (*p* Value, Partial Eta^2^)		0.547 ^b^ (0.024)	0.418 ^b^ (0.041)	0.406 ^b^ (0.016)	0.483 ^b^ (0.015)
Secondary school	58 (52.7%)	58.0 (32.0) ^d^	11.0 (14.0) ^d^	38.5 (8.0) ^d^	26.0 (6.0) ^d^
Vocational post-secondary school	13 (11.8%)	60.0 (26.5) ^d^	13.0 (12.0) ^d^	38.0 (8.0) ^d^	25.0 (8.0) ^d^
University	39 (35.5%)	60.0 (22.7) ^d^	9.0 (12.0) ^d^	40.0 (6.0) ^d^	26.0 (8.2) ^d^
Father’s educational attainment, (*p* Value, Partial Eta^2^)		0.547 ^b^ (0.001)	0.418 ^b^ (0.005)	0.406 ^b^ (0.002)	0.483 ^b^ (0.010)
Secondary school	68 (63.0%)	60.0 (25.0) ^d^	11.0 (11.0) ^d^	38.0 (8.0) ^d^	26.0 (8.0) ^d^
Vocational post-secondary school	12 (11.1%)	50.0 (33.7) ^d^	10.5 (13.0) ^d^	40.0 (3.7) ^d^	27.0 (6.0) ^d^
University	28 (25.9%)	60.0 (31.0) ^d^	7.0 (13.5) ^d^	39.5 (3.7) ^d^	26.0 (8.5) ^d^
Child’s academic performance, (*p* Value, Partial Eta^2^)		0.642 ^b^ (0.023)	0.544 ^b^ (0.010)	0.393 ^b^ (0.002)	0.651 ^b^ (0.029)
Average	6 (5.5%)	41.0 (37.5) ^d^	13.5 (13.0) ^d^	37.0 (17.7) ^d^	26.0 (8.7) ^d^
Above average	40 (36.4%)	60.0 (24.0) ^d^	8.5 (13.0) ^d^	38.0 (9.0) ^d^	26.0 (7.7) ^d^
Excellent	64 (58.2%)	60.0 (28.0) ^d^	10.5 (12.2) ^d^	40.0 (7.0) ^d^	26.0 (7.0) ^d^
Dominant hand, (*p* Value, Partial Eta^2^)		0.177 ^a^ (0.001)	0.136 ^a^ (0.001)	0.875 ^a^ (0.015)	0.841 ^a^ (0.000)
Left	15 (13.6%)	40.0 (36.0) ^d^	7.0 (10.0) ^d^	40.0 (12.0) ^d^	26.0 (12.0) ^d^
Right	95 (86.4%)	60.0 (23.5) ^d^	11.0 (14.0) ^d^	39.0 (7.0) ^d^	26.0 (7.0) ^d^
Sports participation, (*p* Value, Partial Eta^2^)		0.006 **^a^ (0.005)	0.038 *^a^ (0.002)	0.849 ^a^ (0.003)	0.863 ^a^ (0.000)
No	26 (23.6%)	44.0 (42.0) ^d^	5.0 (9.7) ^d^	38.5 (7.7) ^d^	26.0 (6.7) ^d^
Yes	84 (76.4%)	60.0 (22.0) ^d^	11.0 (12.0) ^d^	39.0 (7.0) ^d^	26.0 (6.7) ^d^
Duration of sports participation (months)	60.0 (82.5) ^d^	0.106 (−0.06–0.36) ^c^	−0.027 (−0.20–0.32) ^c^	0.227 (−0.09–0.34) *^c^	0.107 (−0.16–0.28) ^c^
Weekly frequency of sports sessions (hours per week)	5.0 (4.6) ^d^	−0.031 (−0.24–0.20) ^c^	−0.032 (−0.23–0.33) ^c^	0.081 (−0.30–0.15) ^c^	−0.063 (−0.33–0.15) ^c^
Regular attendance at physical education classes, (*p* Value, Partial Eta^2^)		0.931 ^a^ (0.005)	0.139 ^a^ (0.001)	0.438 ^a^ (0.009)	0.836 ^a^ (0.009)
No	3 (2.7%)	60.0 (21.0) ^d^	6.0 (10.0) ^d^	37.0 (9.1) ^d^	28.0 (6.7) ^d^
Yes	107 (97.3%)	60.0 (26.0) ^d^	11.0 (12.5) ^d^	39.0 (7.0) ^d^	26.0 (7.0) ^d^
Body height (m)	1.7 (0.1) ^d^	0.040 (−0.10–5.68) ^c^	−0.152 (−2.86–4.39) ^c^	0.438 (−3.87–2.00) **^c^	0.288 (−4.57–1.57) **^c^
Body mass (kg)	60.6 (13.9) ^d^	0.071 (0.98–14.65) ^c^	0.029 (−7.90–5.49) ^c^	0.364 (−2.27–8.56) **^c^	0.129 (−1.59–9.76) ^c^
BMI (kg/m^2^)	20.8 (4.2) ^d^	0.131 (0.97–6.05) ^c^	0.110 (−2.80–3.55) ^c^	0.169 (−3.59–1.54) ^c^	−0.086 (−5.18–0.20) ^c^
Fat (%)	17.7 (42.5) ^d^	0.116 (2.04–0.28) ^c^	0.106 (−1.61–1.28) ^c^	0.018 (−0.79–1.54) ^c^	−0.166 (−0.37–2.08) ^c^
Muscle (%)	38.1 (8.6) ^d^	−0.148 (2.43–9.61) ^c^	−0.198 (−6.52–8.54) *^c^	0.092 (−1.10–2.08) ^c^	0.228 (−9.11–3.64) *^c^
Bone (kg)	2.9 (0.5) ^d^	0.101 (0.06–2.17) ^c^	0.008 (−1.30–1.35) ^c^	0.427 (−1.53–0.61) **^c^	0.211 (−1.49–0.76) *^c^
Biering–Sorensen test result (s)	140.0 (73.7) ^d^	0.181(0.04–0.41) ^c^	0.068 (−0.26–0.27) ^c^	−0.005 (−0.10–0.32) ^c^	0.088 (−0.14–0.31) ^c^

Notes: N—number of participants; SLS-EO—Single leg stance eyes open; SLS-EC—Single leg stance eyes closed; FRT—Functional reach test; LRT—Lateral reach test; ^a^ Mann-Whitney U test; ^b^ Kruskal Wallis test; ^c^ Spearman’s correlation coefficient (95% CI); ^d^ Median (Interquartile Range); BMI—body mass index. * *p* < 0.05, ** *p* < 0.01.

**Table 2:** Average performance on balance tests in the entire sample and in relation to the age group.

	All Participants (N = 110)	12–15 Years	15–18 Years	*p* Value
SLS-EO (s)	60.0 (28.0) ^a^	44.0 (40.5) ^a^	60.0 (15.5) ^a^	0.002 ^b^
SLS-EC (s)	10.0 (12.2) ^a^	9.0 (13.0) ^a^	10.0 (12.0) ^a^	0.605 ^b^
FRT (cm)	39.0 (7.0) ^a^	39.0 (9.0) ^a^	39.0 (7.0) ^a^	0.459 ^b^
LRT (cm)	26.0 (6.2) ^a^	28.0 (5.5) ^a^	25.0 (7.0) ^a^	0.007 ^b^

Notes: N—number of participants; SLS-EO—Single leg stance eyes open; SLS-EC—Single leg stance eyes closed; FRT—Functional reach test; LRT—Lateral reach test; ^a^ Median (Interquartile Range); ^b^ Mann-Whitney U test.

**Table 3:** Predictors of balance performance among the participating adolescents.

	SLS-EO Beta [95% CI]	SLS-EC Beta [95% CI]	FRT Beta [95% CI]	LRT Beta [95% CI]
Sex (ref.: Female)				
Male	−0.120 [−11.63, 2.63]	−0.103 [−6.37, 1.87]	0.168 [−0.23, 4.12]	0.206 [0.19, 4.00]
Age (years) (cont.)	0.247 [0.75, 5.20] **	−0.084 [−1.90, 0.74]	0.072 [−0.44, 0.97]	−0.219 [−1.32, −0.11] *
Duration of sports participation (cont.)	0.201 [−0.05, 0.16]	0.014 [−0.05, 0.06]	0.173 [−0.06, 0.05]	0.020 [−0.02, 0.03]
Weekly frequency of sports sessions (cont.)	0.032 [−0.98, 1.32]	−0.047 [−0.92, 0.59]	0.071 [−0.26, 0.52]	−0.074 [−0.48, 0.24]
BMI (cont.)	0.005 [−1.06, 1.11]	0.010 [−0.59, 0.66]	0.156 [−1.06, 0.60]	−0.069 [−0.40, 0.19]
Fat (%) (cont.)	0.068 [−0.28, 0.59]	0.062 [−0.17, 0.33]	−0.022 [−0.15, 0.12]	−0.177 [−0.23, 0.01]
Muscle (%) (cont.)	−0.089 [−1.18, 0.43]	−0.123 [−0.76, 0.16]	0.131 [−0.08, 0.42]	0.237 [0.06, 0.48] *
Bone (kg) (cont.)	−0.134 [−2.39, 13.97]	−0.058 [−6.21, 3.30]	0.444 [3.62, 8.17] **	0.162 [−0.30, 4.11]
Biering–Sorensen test result (cont.)	0.224 [0.01, 0.13] *	0.075 [−0.02, 0.05]	−0.036 [−0.02, 0.15]	0.014 [−0.01, 0.02]

Notes: SLS-EO—Single leg stance eyes open; SLS-EC—Single leg stance eyes closed; FRT—Functional reach test; LRT—Lateral reach test; BMI—body mass index. * *p* < 0.05, ** *p* < 0.01.

## Data Availability

The data presented in this study are available on request from the corresponding author. The data are not publicly available due to the protection of personal data.
